# Valedictory Address to the Graduates in Comparative Medicine and Veterinary Science of McGill University, Montreal

**Published:** 1896-05

**Authors:** Wesley Mills

**Affiliations:** Professor of Physiology in the Faculty of Human Medicine and in the Faculty of Comparative Medicine and Lecturer on Cynology in the Latter Faculty


### THE JOURNAL
OF
### COMPARATIVE MEDICINE AND VETERINARY ARCHIVES.

Vol. XVII. *MAY*, 1896. No. 5.

### VALEDICTORY ADDRESS TO THE GRADUATES IN COMPARATIVE MEDICINE AND VETERINARY SCIENCE OF McGILL UNIVERSITY, MONTREAL.

By WESLEY MILLS, M.A., M.D., D.V.S.,
PROFESSOR OF PHYSIOLOGY IN THE FACULTY OF HUMAN MEDICINE AND IN THE FACULTY OF COMPARATIVE MEDICINE AND LECTURER ON CYNOLOGY IN THE LATTER FACULTY.

To-day we, your teachers, congratulate you on having overcome, by your industry and perseverance, the obstacles that have been in your path during the past three years. Having now reached the goal, you may honestly rejoice, and if your bosoms swell with pride, it is, we know, a pride tempered by the conviction that the mental status to which you have attained is very far removed from that which you hope to reach by the same wise use of your powers in the years that are to come. We and you alike realize that this day marks an era. Henceforth you walk your way without any man's special guidance. You take your fates in your own hands. You are very different men, to-day, from what you were three years ago. Were it not so, you would have failed to pass those examinations conducted both by your teachers and that independent board composed of practising members of your own profession, by virtue of which this University has conferred upon you the degree of Doctor of Veterinary Science.

As the choice of a calling or profession determines to a large extent the manner in which the individual shall use his powers during his whole life, it is for himself and his fellows, necessarily, one of the most important decisions of a lifetime.

In fact, it is only excelled in gravity by the choice of one's principles, and one's partner for life. The man of no fixed principles of action is like a ship without a compass or rudder; the man of bad principles is a constant menace to society. The individual who selects either profession or principles at haphazard sins against himself and his fellows; but he who chooses lightly her who is to determine the future, does so still more, for the laws of heredity are as certain and inviolable as any others, and heredity is of infinitely more importance in the large proportion of cases than environment. I hope, therefore, that this latter choice has been delayed till it can be made with an insight into the laws of Nature you once did not possess.

Probably no body of young men chooses a profession more from a love of it than students of comparative medicine. It is rare to find a candidate who is not an ardent admirer, to say the least, of some variety of our domestic animals, while many have for our dumb fellow-creatures the keenest sympathy and no mean appreciation of their natural qualities. In this they can claim affinity with some of the noblest of mankind of both sexes. It is a matter of history that not a few of the best, most highly endowed, and most distinguished of our race have recognized their fellowship with our speechless servants, pets, and friends; while wilful neglect and cruelty are certain marks of a mean and ignoble nature. The man who kicks his horse or his dog is only too prone to abuse his wife and children, or grind down his fellow-men.

But, now that you leave college halls for the larger world, the view you take of your profession, both as to what it is and what it may become, is of supreme importance.

It so happens that, just at the present time, there are, apparently, some special grounds for discouragement. The clatter of horses' feet in the streets of cities has been in no small degree replaced by the electric motor and the noiseless speeding wheel of the bicyclist, and the great horse-ranches and breeding-farms have in many cases ceased to exist; but the pork-barrel is still being filled, and butter, cheese, and milk have not been banished from our tables; people, as yet, prize woollen garments above all others; boots and shoes continue to be made from the prepared skins of our domestic animals, and all require them; the broad pastures of this great continent are dotted over with fleecy sheep, and the cattle on a thousand hills have a utilitarian as well as a poetical significance; the hungry millions of Europe await our supplies of meat, and I only wish that we could put some into every expectant mouth.

It is scarcely likely that that animal which has played so conspicuous a part in domestic service, in the chase, in war, in romance, and on the course will cease to grace the earth and to delight mankind. I am inclined to believe, however, that the present subordination of the horse, with the consequent necessary decrease in equine medical practice, is a blessing in disguise. It has always seemed to me unfortunate, both for man and beast, that the veterinarian's education and practice were so largely confined to the horse. In reality the interests of the mass of mankind are more closely bound up with the well-being of other animals. We are all dependent on them for our necessaries, even our very food; and it is becoming more and more apparent with the progress of medicine that the health of mankind is in large measure determined by that of our domestic animals. They are liable to many of the same diseases as ourselves, and that malady—plague, it might almost be called—which still carries off at least one-tenth of the whole human family is frightfully prevalent in that very class of animals on which we are most dependent, viz., the bovine species. In the domestic cow, almost a part of the family, there may lurk hidden beyond the power of detection, except by recently discovered means, the germs of that disease, tuberculosis, of all the most widespread and destructive to the human family, and from this animal, the family or dairy cow, there is now no longer any doubt that it can be, and is, conveyed to man.

The public is at last, thanks to the efforts of medical men and the diffusion of knowledge by the printing press, aroused to the gravity of the danger that menaces every family. Loving mothers and anxious fathers bending over the cradles of their sick children, with a suspicion as to the cause of the evil, and a determination as to the remedy, demand that those on whom the public responsibility rests shall see to it that milk is not only free from added water, but from deadly germs; and they are generous enough to insist that the benefits shall not be confined to their offspring, but extend to the children of the ignorant poor, who, though unaware of the cause of the dreadful scourge, none the less suffer the pangs of bereavement.

An intelligent public will see that inspection must extend to dairies and to the cattle themselves, as well as to the milk and to the butcher's meat. There is but one class of men—veterinary surgeons—properly qualified to carry out such inspection. There should be no half-measures, no temporizing, no dallying with these most imperative reforms, in comparison with which a large proportion of the subjects that engage the attention of the representatives of the people are of trifling importance. And this is but one illustration, among many, of the relations the diseases of mankind and of the lower animals sustain to one another.

The whole science of medicine and surgery is undergoing radical changes—one might almost say, being revolutionized. The results of the noble devotion to the pursuit of knowledge, for its own sake and because of its bearing, mostly not obvious at the time, on the welfare of mankind, by the eminently gifted of different lands, well represented by the late illustrious Pasteur, are in these days being turned to practical account to ward off disease and death. Plagues such as once devastated Europe are no longer possible anywhere among civilized communities, and we have now good reason to hope that, one after another, the known infectious diseases will be prevented or overcome, both among men and beasts. And how is this being brought about? Chiefly by experiments on the lower animals. And yet we hear, in some quarters, the senseless cry of antivivisection. Experiments on the lower animals, involving as they must, in some instances, vivisection, would be well worth while, even if only his inferiors, and not man at all, were concerned. Every advance in preventive and restorative medicine benefits, or should benefit, both man and beast. It follows from all this that the scope of medicine has vastly widened; that its progress has, of late, been extremely rapid; that its powers for good are greatly enlarged; that the outlook is most hopeful, and that in all this we have reason to believe that man and beast will, alike, be participators if enlightened views as to the nature, rights, and relations of the lower animals prevail.

It must, therefore, follow, that the profession of medicine is greater in its importance to-day than ever before. The temporary subordination of the horse may prove a blessing in disguise, if the students of comparative medicine are led to devote their energies to a more thorough investigation of the nature of our other domestic animals, both in health and in disease. All the great advances in medicine, those most affecting the financial and the domestic interests of mankind, those appealing both to the pocket and the heart, have been effected through a study of the lower animals. It is no longer possible to recognize two entirely distinct branches of the science of medicine. Medicine is, as applied to man, no longer a system of blind empiricism, nor, as applied to the lower animals, a combination of that with farriery. The barber-surgeon and the farrier are but landmarks in the history of the evolution of medicine. Gentlemen, there is but one animal kingdom, governed by the same natural laws, applicable alike to man and his fellow-creatures, lower, in some respects, in the scale, but sharing with him the liability to disease and death. If this be so, the only true best method of investigating disease, as, indeed, everything else, is the comparative method.

Comparative medicine is the medicine of the future, and the sooner that is realized the better for man as well as beast. Indeed, we now grasp that future—the present touches its skirts. Specialism, or division of labor, will be necessary, because the powers of individuals are limited. Some will elect to treat the lower animals, and some mankind, with even further subdivision; but there is only one science and art of medicine; and all the various bodies of workers in this vast field should form but different battalions of one great army fighting for the prolongation of vigorous life and the mitigation of pain in every quarter to which the power of man can reach; and heaven forbid that any erroneous notions or false pride, any mere snobbery, should stand in the way of the great objects to be attained! I recall the fact that it was on this very day of the month, seven years ago, that Dr. R. P. Howard, the late distinguished and revered dean of the faculty of human medicine, passed from the scenes of his busy life. It may not be generally known that it was one of the long-cherished hopes of his life to see established in this University a chair of comparative pathology. Nothing, to my mind, could better have demonstrated the remarkable insight of the man. He, like other great men, dreamed dreams and saw visions. When shall we behold these realized in the establishment of one great, broad school of medicine in this University, which shall exist for the purpose of investigating all forms of disease to which living things are liable—their causes, their prevention, their cure?

Gentlemen, what a great profession is yours! What grand possibilities, what a glorious outlook! How the prospect fills the mind and satisfies the idealistic longings of the enlightened and aspiring young man! I congratulate you on the choice of such a profession. I do not say that the medical is the grandest of all professions. To my mind there is no best profession, but that is best for which the man is the most perfectly suited, and there is just so much nobility in each man's career as he puts into it. But what more can you wish than to behold in your profession the possibilities of unlimited good for the whole sentient creation? Such a profession calls, however, for men of no mere smattering of knowledge, no ordinary aims, no easily satisfied ideals. Of such people there are more than enough in all the professions already. It is unnecessary to remind you that your education is but fairly begun. Take the earliest opportunity of quitting the routine of practice, and, betaking yourselves to some institution where the best men and the most perfect equipment are to be found, and again become earnest investigators, under more favorable conditions than an ordinary practice affords.

I would that I could truthfully say that we were now prepared to offer you, in a post-graduate course here at McGill, the best to be obtained in America, or in the world. I doubly wish that this were so when I call to mind the fact that the dean of this faculty of comparative medicine has given the best thirty years of his life to the struggle to maintain in Canada, and in Montreal, a high standard of veterinary education, and that, too, at a time when other schools were content with very, very much less than the Montreal Veterinary College (as it was then known) required. The present lack of suitable buildings and equipment, discouraging to all of us, must be doubly so to him. May some generous friend, grasping the conception of the importance of comparative medicine, seize the opportune moment to make for himself and this faculty a name for large and permanent usefulness.

But, gentlemen, to you, individually, let me say, men are more than means. The mind can rise above environment, and the greatest of our race have been those that did so. The book of Nature is open to you, as to others, and it is not in the power of the rich to shut it, though they can do much to make opening easier. You may not be able at first to afford many books, or much time for elaborate investigation, but you will have daily opportunities for observation, and you can masticate well the mental food that comes to hand. Good digestion, assimilation, and growth will follow. Consort with the best in the medical profession, no matter in what direction their powers are applied. Read the soundest literature, as far as it is accessible to you, whether of the human or veterinary branch of medicine, but, above all, observe, try, test, investigate for yourselves. In that way alone will you attain to the highest, healthiest mental life. Only thus can you add to the sum of knowledge. Oh! how vast is the field, how untilled, as yet, and how few the laborers that are prepared to do the highest kind of work!

But it is not alone in connection with the higher work of the profession that opportunities of usefulness are to be found. Even the most ordinary practice offers to the veterinarian scope for bettering the condition of man and beast, and in ways somewhat different from those to which reference has already been made. To the man of means the loss of an animal may signify little more than a slight reduction in the amount of his wealth. But to the poor man, dependent on his animal for his very livelihood, its sickness must mean grave anxiety, and its death, possibly, serious embarrassment; so that it may be in your power not only to relieve the animal, but to remove a load of anxiety from the owner's mind and, perchance, to avert impending calamity.

Demand and compel adequate remuneration for your services from those who are able to pay, but refuse not the helping hand to the poor because there is no prospect of pecuniary reward. Pull the ox and the ass out of the pit, though they belong to the poor, or even to no man, so far as you may know, for by so doing you will help the helpless and minister to your own better nature.

Gentlemen, it will be your privilege to diffuse a truer knowledge of the laws of life, of the conditions that make for health and disease, to suggest methods for the improvement of the breeding and rearing of a better class of animals and to aid in the prevention of thoughtless cruelty. Allow me to express the hope that you and the other graduates in comparative medicine, who have had special training in studying the psychic nature of animals, will endeavor to interest those you may meet in this aspect of animal existence, and to help the public to realize that those animals by which we are daily surrounded are, in reality, our fellows, with not only a like susceptibility to suffering, but with not wholly dissimilar feelings and intelligence. Even the dullest of them have more within the round of their lives than we usually realize.

But what shall we say of those more intelligent creatures, the horse and the dog? From time immemorial the dog has been, in one sense or another, the companion of man. He has, by countless thousands, been recognized as the guardian of property, the defender of women and children, the amusing pet and the ever-willing servant. He enters into our every mood and readily adapts himself to our caprices. Grateful for the smallest favors, even a kindly word, he nevertheless accepts kicks and blows, however undeserved, and loves still him who has forfeited all claim to affection. He willingly shares in his master's poverty or degradation, and when forsaken by all men he will not leave him, even though he perish in the gutter, and he will guard his outcast form till he draws his own last breath. What a rare combination of qualities in the dog! How much poorer the world would be without him! But in all our domestic animals there is much to admire, if we but properly understand them; and it does seem to be no unworthy use of time and energy to make their real nature better known.

It may be that, by such services as those to which I have been alluding, you will not put money in your purse, but you will be laying up for yourselves treasure in the heaven of your own breasts. But, gentlemen, the time would fail me to do justice to the greatness of your profession. We, your teachers, all wish you well, and what better can we desire for you than that you may be good men and true, seeing in your chosen calling and in life what is and what may be, and that, inspired by worthy ideals, you may live noble lives. Farewell.

